# Periodic Motion in the Chaotic Phase of an Unstirred Ferroin-Catalyzed Belousov Zhabotinsky Reaction

**DOI:** 10.3389/fchem.2022.881691

**Published:** 2022-07-08

**Authors:** Florian Wodlei, Mihnea R. Hristea, Giuseppe Alberti

**Affiliations:** Living Systems Research, Klagenfurt, Austria

**Keywords:** BZ reaction, ferroin-catalyzed, chaotic transient, periodic patterns, convection cells

## Abstract

The Belousov Zhabotinsky reaction, a self-organized oscillatory color-changing reaction, can show complex behavior when left unstirred in a cuvette environment. The most intriguing behavior is the transition from periodicity to chaos and back to periodicity as the system evolves in time. It was shown that this happens thanks due to the decoupling of reaction, diffusion and convection. We have recently discovered that, as the so-called chaotic transient takes place, periodic bulk motions in form of convective cells are created in the reaction solution. In this work we investigated this phenomenon experimentally by changing cuvette size and reaction volume, in order to allow different types of convection patterns to appear. So far, we have observed single and double convection cells in the system. There are indications that the convection patterns are connected to the duration of the chaotic phase. A simplified mathematical model confirms the form and dynamics of the observed convection cells and explains the connection between chemical chaos and hydrodynamical order.

## Introduction

The Belousov Zhabotinsky (BZ) is a typical example of a chemical oscillator that shows self-organizing behaviour at different levels. It is the most thoroughly studied oscillatory reaction system in homogeneous phase in chemistry. At the same time, due to its complexity, not all aspects of its dynamics are yet understood. After its discovery by Boris P. Belousov (Belousov, 1959) in the 1950s and the thorough investigation by Anatol M. Zhabotinsky and other colleagues (Zhabotinsky, 1964; Korzukhin Zhabotinsky, 1965; Zaikin and Zhabotinsky, 1970) it became famous in the 70s where major achievements were gained in trying to understand its reaction mechanism (Field et al., 1972; Noyes et al., 1972; Field and Noyes, 1974a; Field and Noyes, 1974b).

The BZ reaction is a periodic bromination of an organic species in acidic environment and in the presence of a catalytic color indicator in liquid phase. Convection and diffusion play an important role in liquid phase systems. It is possible to observe beautiful periodic color oscillations when the reaction is carried out in well stirred batch reactor to hinder diffusion and convection. The formation of chemical waves can be observed in a Petri dish where the development of convective motion is suppressed.

The BZ reaction is not the only chemical oscillator but the first found in homogeneous phase (Scott, 1993; Tyson and Levin, 1994). Subsequently, many other systems, showing similar oscillatory and self-organizing properties, were discovered in both biology and chemistry. In biology the majority of oscillatory reactions are related to the so called biological clocks, for example, those that occur during the cell cycle (Chance et al., 1973; Goldbeter, 1996). For instance, an interesting system in biology is that of the Min protein system found in *E. Coli*. This system ensures the proper spatial and temporal regulation of chromosomal segregation and division prior to cell division. The Min proteins spontaneously form chemical surface waves on artificial flat membrane *in vitro* (Loose et al., 2008) that show high similarity to those observed in the BZ reaction. Nowadays in chemistry we know many systems that show oscillatory and self-organizing properties. In particular, the cetrimonium bromide (CTAB)/dichloromethane system shows especially beautiful self-organization. More specifically, it is a biphasic system where oscillations of the surface tension occur at the interface between CTAB and dichloromethane (Pimienta et al., 2004). Even though their origin is different, they show a surprisingly similar shape to the ones seen in a BZ reaction. When only one little drop of dichloromethane is added to a CTAB solution, it is possible to observe highly organized patterns, such as oscillations of the drop diameter (Antoine et al., 2016; Wodlei et al., 2018). As in the BZ reaction, convection plays a crucial role in this system too.

Such similarities are no coincidence; rather they are the fulfilment of certain conditions that allow periodic behavior to occur. Irving Epstein and colleagues, for example, were actively searching for such oscillators and successfully constructed one following these conditions (Epstein and Pojman, 1998).

At present, researchers employ the BZ reaction not only to investigate and better understand its dynamics, but for many different applications. It is used in elastomechanical gels to generate periodical motion (Deb et al., 2014), to create binary operations in non-convectional computing (Adamatzky and Costello, 2007; Adamatzky, 2019), to mimic neural dynamics (Gentili et al., 2017) and to implement chemical automata to mention only a few (Dueñas-Díez and Pérez-Mercader, 2021).

In this study we investigate the dynamics of the BZ reaction when left unstirred in a batch reactor. Due to the fact that the system is liquid and unstirred, its dynamics will be not only governed by the reaction kinetics, but also by diffusion and convection. In this case the reaction dynamics becomes more complex. In addition to the periodic color change, a phase of aperiodic color change appears for a limited time that transits back to periodicity. The appearance of this chaotic transient led some researchers to even call this behavior a “re-birth” (Onuma et al., 2011), even though the system never reaches thermodynamic equilibrium during the chaotic phase. More specifically, the reaction system is undergoing a transition to an aperiodic phase from which it returns to periodicity after several hours. This behavior was first discovered by Rustici et al. in a Cerium-catalyzed (Rustici et al., 1996) and later also in a Ferroin-catalyzed version (Rossi et al., 2009) of the BZ reaction. Rustici et al. (Rustici et al., 1998) also demonstrated that the transition to chaos follows a Ruelle-Takens-Newhouse (RTN) scenario (Newhouse et al., 1978).

An unstirred BZ reaction behaves in a more complex manner due to the fact that convections of different origins are not hindered by the homogenizing effect of stirring and are free to develop instead. Therefore the resulting dynamics is an interplay between diffusion, convection and (local) kinetics.

Chaotic behavior is not found only in unstirred batch reactors but also in open constantly stirred tank reactors (CSTR), where the reaction solution flows in and out with a specific flow rate. In this case, the appearance of chaos is totally dependent on the flow rate. High flow rates are connected with chaos (Gyorgyi et al., 1992).

The appearance of chaos in an unstirred BZ reaction, on the other hand, shows strong relations to the complex coupling that happens in this system between chemical kinetics, diffusion and convection. The effect of convection on the chaotic phase was demonstrated by Budroni et al. (2017) by varying the solution viscosity. An increased viscosity hinders the formation of convection; the duration of the chaotic phase in such systems could be reduced, even until the complete disappearance of the chaotic transient. It was proven that the temperature has an effect too (Masia et al., 2001). Variation in temperature, between 0 and 8°C, leads the system from periodicity to chaos. Thus, temperature affects the coupling between chemical kinetics, diffusion and convection in a similar way. Marchettini et al. (2010) theoretically showed that the consumption of the chemical species is at the origin of the decoupling of reaction kinetics, diffusion and convection.

Our group discovered that this unstirred dynamics can also be affected by applying a limited stirring phase immediately following the first periodic phase. By doing so, the duration of the chaotic phase is shortened (Wodlei and Hristea, 2013).

Convective motion in the BZ reaction was mainly investigated in the shallow configuration, in order to study the spatial patterns by varying, for example, the layer depths (Rossi et al., 2012). To study the temporal dynamics, either constantly stirred tank reactors (CSTR) or stirred standard cuvettes were used. Due to the homogeneity of the reaction solution the system is usually examined with spectrophotometers. Even though the homogeneity in an unstirred batch configuration is not always given, the majority of the studies carried out were conducted in standard photometric cuvettes and investigated by using spectrophotometers. So far, to our knowledge, convective motion in the BZ reaction was never studied in a cuvette configuration. This might be due to the fact that the convection dynamics in a filled standard cuvette shows only very slow upwards and downwards motions, hardly visible to the eye. However, convective motion was observed in another system, in a cuvette configuration. Gentili et al. investigated color oscillations and waves due to hydrodynamic convective motion in a solution of a thermoreversible photochromic spiro-oxazine (Gentili and Micheau, 2019).

## Experimental Methods

**FIGURE 1:**
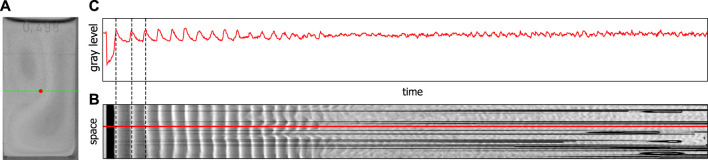
Extraction of time series from video data, **(A)** shows a video image of the BZ reaction in a special cuvette, **(B)** shows a space–time plot for the green cross-section line in **(A), (C)** shows the gray level value profile along the red line in **(B)** corresponding to the change in gray level at the red point shown in **(A)**.

A BZ reaction in a cuvette configuration is usually investigated in a spectrophotometer. A concentrated light beam is sent through a point in the middle of the cuvette and the absorbance/transmission is registered by a photo diode or another type of sensor. Such measurements can give great insight into the dynamics that occur in homogeneous reaction solutions. A constant stirring of the solution does usually guarantee its homogeneity. In an unstirred BZ reaction such a homogeneity is generally not achieved, and therefore a spectrophotometric analysis can only provide a certain amount of insight into the reaction dynamics. To record the entire cuvette we used a video camera instead of a spectrophotometer, which allowed us to record the spatial dynamics of an unstirred BZ reaction. Additionally, we could extract from the video data time series by creating space–time plots from cross-section lines along the width of the cuvette. Since we are interested in the dynamics of a given point (see Figure 1A, red point) which corresponds to a line in the space-time plot (see Figure 1B, red line) we translated the shades of gray back into gray level values and obtained a time series (see Figure 1C). These time series are similar to those obtained by a spectrophotometer. Due to the sensitivity of the sensor chip of the video camera these time series correspond to a wavelength of around 450 nm. For more details, see the Supplementary Material S1.

### Materials and Instruments

**FIGURE 2:**
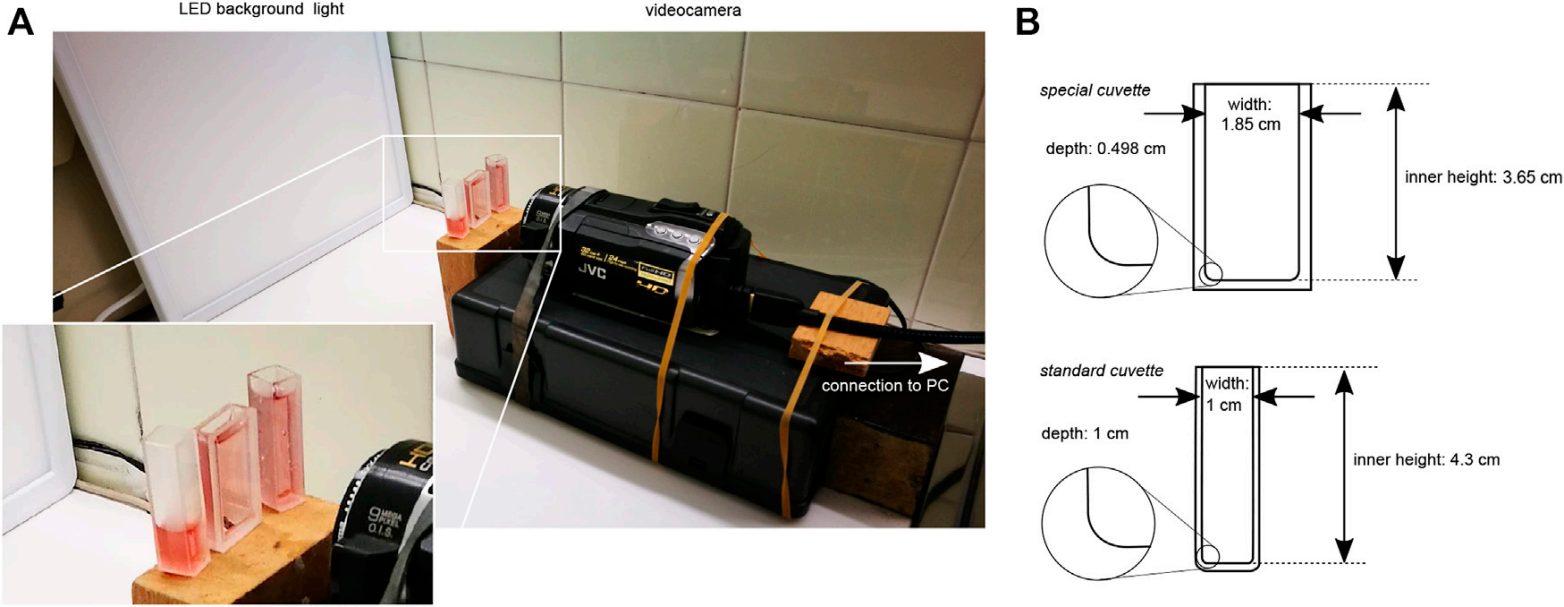
Optical setup, **(A)** shows the arrangement of the LED backlight, photo spectroscopic quartz cuvettes and the video camera; inset shows a zoom on the cuvettes, **(B)** shows the schemes of the cuvettes with their dimensions; insets show the curved inside corners.

An optical setup consisting of a ceiling LED panel (B.K.Licht 18 W LED Panel, 2,400 lm, 4000 K white light) as background light and a video camera (JVC Everio HM400) was used to record the dynamics of the reaction. The filled cuvettes of different types were placed in the focal point of the camera in front of the backlight (see Figure 2).

### Preparation of the Solution

All chemicals were commercial grade reactants (Sigma-Aldrich) and were used without further purification. All solutions were prepared with laboratory reagent grade distilled water. The four following aqueous stock solutions with their specific concentrations were prepared by weight, using an analytical balance: malonic acid (MA) 2 M, sodium bromate (NaBrO_3_) 1 M, sulfuric acid (H_2_SO_4_) 2 M, sodium bromide (NaBr) 1 M. The ferroin solution with a concentration of 0.025 M was used without further dilution.

The reaction solution was prepared by adding the following volumes of the stock solutions and of distilled water with a micropipette, in a volumetric flask: 1.5 ml distilled water, 1.265 ml MA solution, 0.675 ml NaBrO_3_ solution, 3 ml H_2_SO_4_ solution, 0.12 ml NaBr solution and 0.1 ml ferroin solution. The resulting solution then was stirred for 15 min at a high stirring rate before being poured into the spectrophotometric quartz cuvettes of different volumes and geometries. All experiments were performed at a constant room temperature of 22°C.

## Experimental Results and Discussion

**FIGURE 3:**
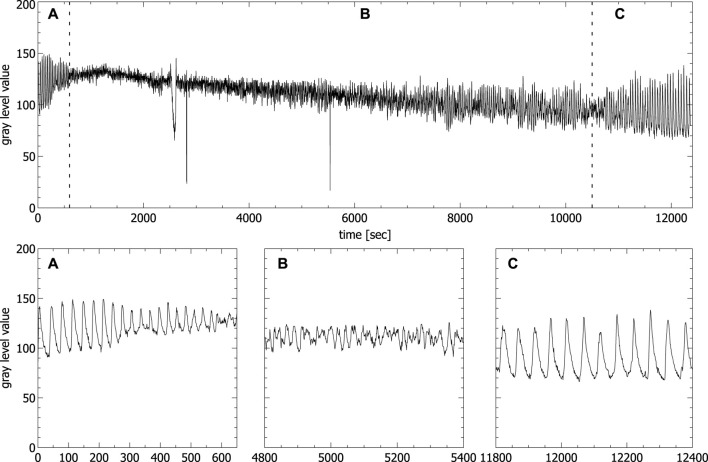
Oscillations of the concentration of the ferriin concentration extracted from video data of an unstirred BZ reaction carried out in a fully filled standard cuvette (spikes in the time series correspond to the passage of bubbles). Panels **A–C** show the enlargement of the areas **A–C** in the upper part corresponding to the first periodic **(A)**, the chaotic **(B)** and the second periodic phase **(C)**, respectively.

Figure 3 shows a typical time series of a ferroin-catalyzed BZ reaction in unstirred batch conditions, obtained from video data of an open, 3 ml filled standard quartz cuvette (1 × 1 × 4.5 cm). Similar time series produced by spectrophotometric measurements of unstirred BZ reaction in unstirred batch conditions were also obtained by others (Rustici et al., 1996; Rossi et al., 2009; Marchettini et al., 2010).

The typical transition from a region of periodic (see Figure 3A) to a region of aperiodic oscillations (see Figure 3B) and back again to periodic oscillations (see Figure 3C) is clearly visible. Only the beginning of the second periodic phase is shown, since our interest is in the aperiodic phase. The second periodic phase itself can last up to 8 h before the reaction reaches thermodynamic equilibrium. It has been shown that the phase of aperiodic oscillations shows two distinct features of deterministic chaos, namely a broadband spectrum in the frequency domain and positive maximal Lyapunov exponents (Rossi et al., 2009). We have analyzed the aperiodic regime of the time series shown in Figure 3, obtained from video data and we also got a broadband spectrum in the frequency domain and a positive maximal Lyapunov exponent calculated with the TISEAN Nonlinear Time Series Analysis package (Kantz and Schreiber, 2004), comparable to the one obtained by Rossi et al. (Rossi et al., 2009) (for details, see Supplementary Material S1). For that reason, from now on we will name the region of aperiodic oscillations *chaotic* phase.

The results reported here were obtained from a series of seven experiments conducted under the same conditions. For each experiment the BZ reaction solution was prepared as described above and poured into two standard cuvettes (1 × 1 × 4.5 cm), one partially filled (1.3 ml), the other one fully (3 ml), and one fully filled (3 ml) slimmer but broader special cuvette (1.85 × 0.498 × 3.65 cm) that were all already placed in a simple optical setup (see Figure 2).

Even though the same conditions were used for all the experiments, slight unavoidable variations in the initial conditions of the concentrations and the temperature led to small differences in the evolution of the reaction. The observed oscillation period of the first periodic phase is the same in all cuvettes from a given mother solution independent of the reaction volume or shape (e.g. 35.33 ± 0.5 s), while the oscillation period between different mother solution with the same apparent initial conditions can vary up to 30% (i.e. 35.33 s ± 0.46 s vs. 25.48 ± 0.16 s). This variation can be understood in terms of the uncertainty of the micropipette that leads to an uncertainty in the concentrations of around 1% and temperature variation of maximal 1°C, knowing that the oscillation period depends on the initial concentration of the reactants and the temperature. The observed convection patterns are also sensitive to these slight variations, even though the general dynamics is unaffected by them.

All the experiments conducted were accompanied by a “bubble phase” that started soon after pouring the substances in the cuvettes and lasted between 30 and 40 min. In this phase bubbles formed at the glass walls of the cuvettes and moved upwards when they reached a certain size. These gas bubbles are composed of carbon monoxide and carbon dioxide (Onel et al., 2007). The additional volume created by the bubbles led to an apparent volume increase of the reaction solution, following by its decrease after the most of the bubbles were released. Due to this formation of gas during the whole duration of the reaction the cuvette could not be closed as it would have been necessary to avoid evaporation.

**FIGURE 4:**
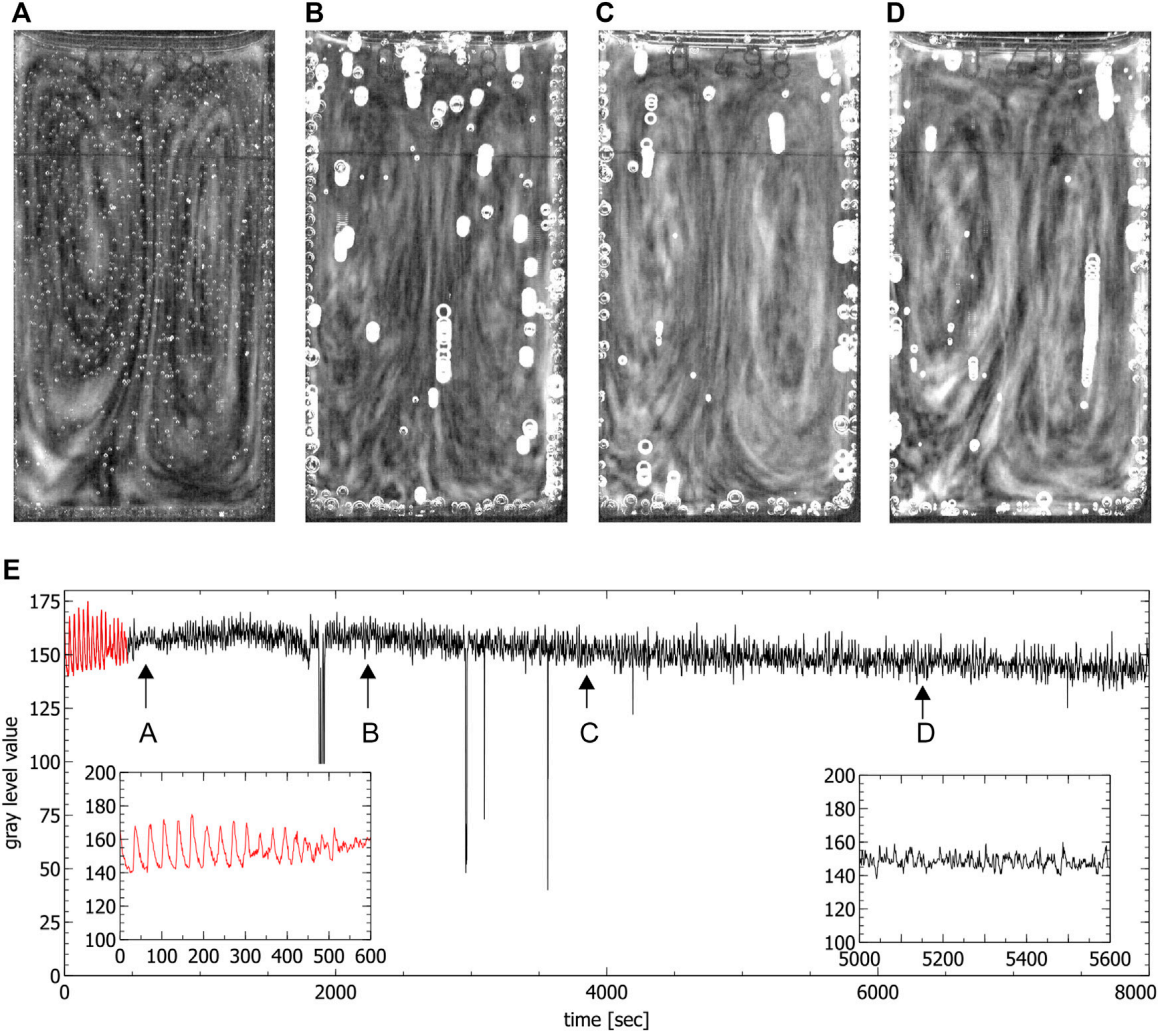
Convection cells in the special cuvette during the chaotic phase. **(A–D)** show the presence of two convection cells (liquid moves up in the middle and down on the left and right side). Images were obtained by averaging over 60 s, **(E)** shows the time series extracted from the video data where the red part corresponds to the periodic phase. Arrows indicate the times where the images **(A–D)** were created (spikes in the time series correspond to the passage of bubbles). Insets show the periodic phase (left) and part of the chaotic phase (right) magnified.

During the chaotic phase ordered convective motions were visible in the partially filled standard cuvettes, where one convection cell formed (see Figure 6) and in the special cuvette, where two convection cells formed (see Figure 4). A first estimation of the magnitude of the convection velocity gives a value range of 0.5–0.6 mm/s (calculated in the experiment shown in Figure 4). The convection cells stayed stable throughout the whole chaotic phase. Only in some of the experiments conducted in the special cuvette one of the two convection cells became more dominant while the second one got reduced in size. This situation was not stable and there was a continuous switching between two convection cells of the same size and two of different sizes.

**FIGURE 5:**
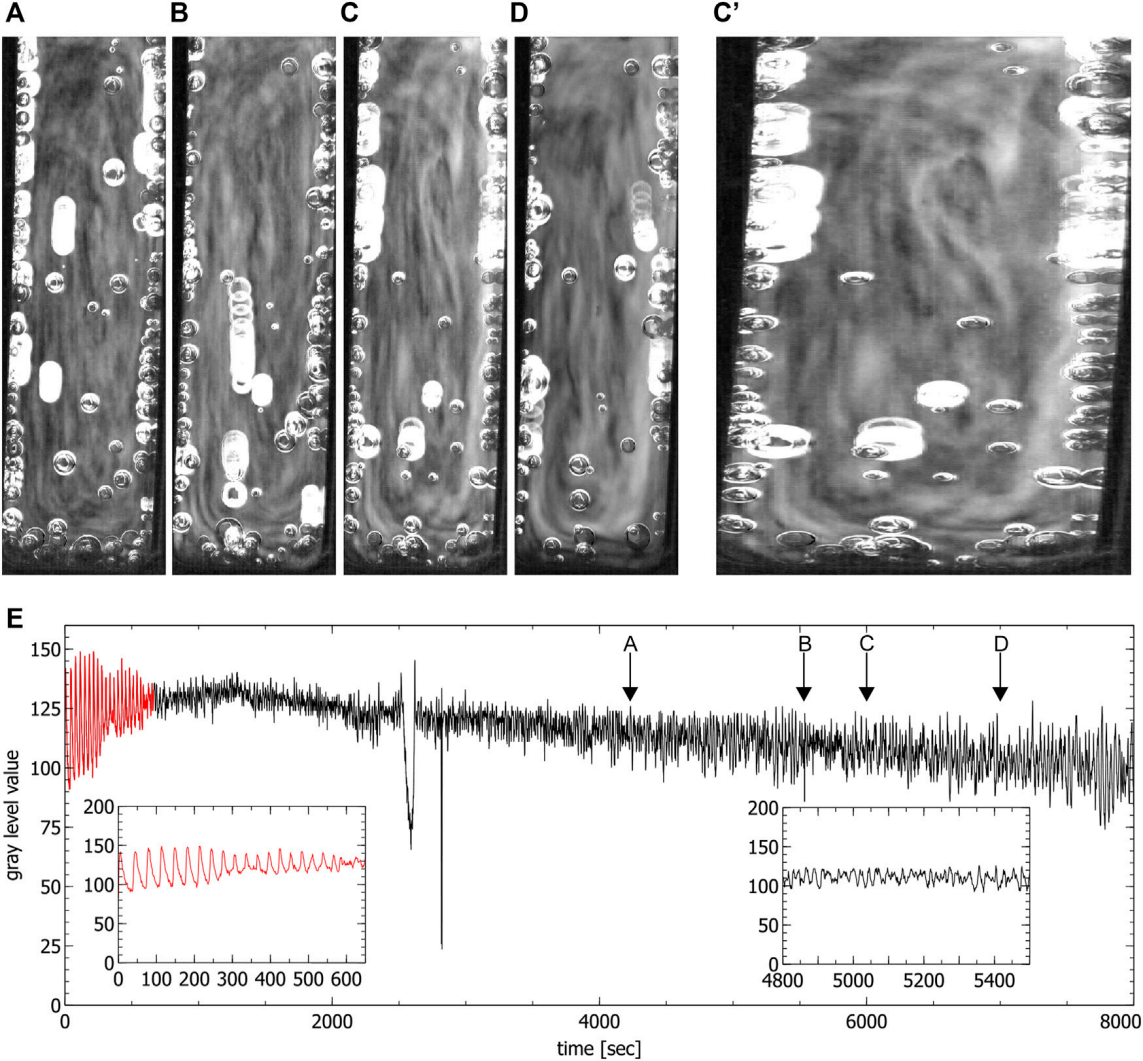
Convection cells in a fully filled standard cuvette during the chaotic phase. **(A–D)** show the up and down motion inside the liquid. Images were obtained by averaging over 60 s **(C’)** shows subfigure **(C)** stretched for better visibility, **(E)** is the time series extracted from the video data where the red part corresponds to the periodic phase. Insets show the periodic phase (left) and part of the chaotic phase (right) magnified (spikes in the time series correspond to the passage of bubbles). Arrows indicate the times where the images **(A–D)** were created.

**FIGURE 6:**
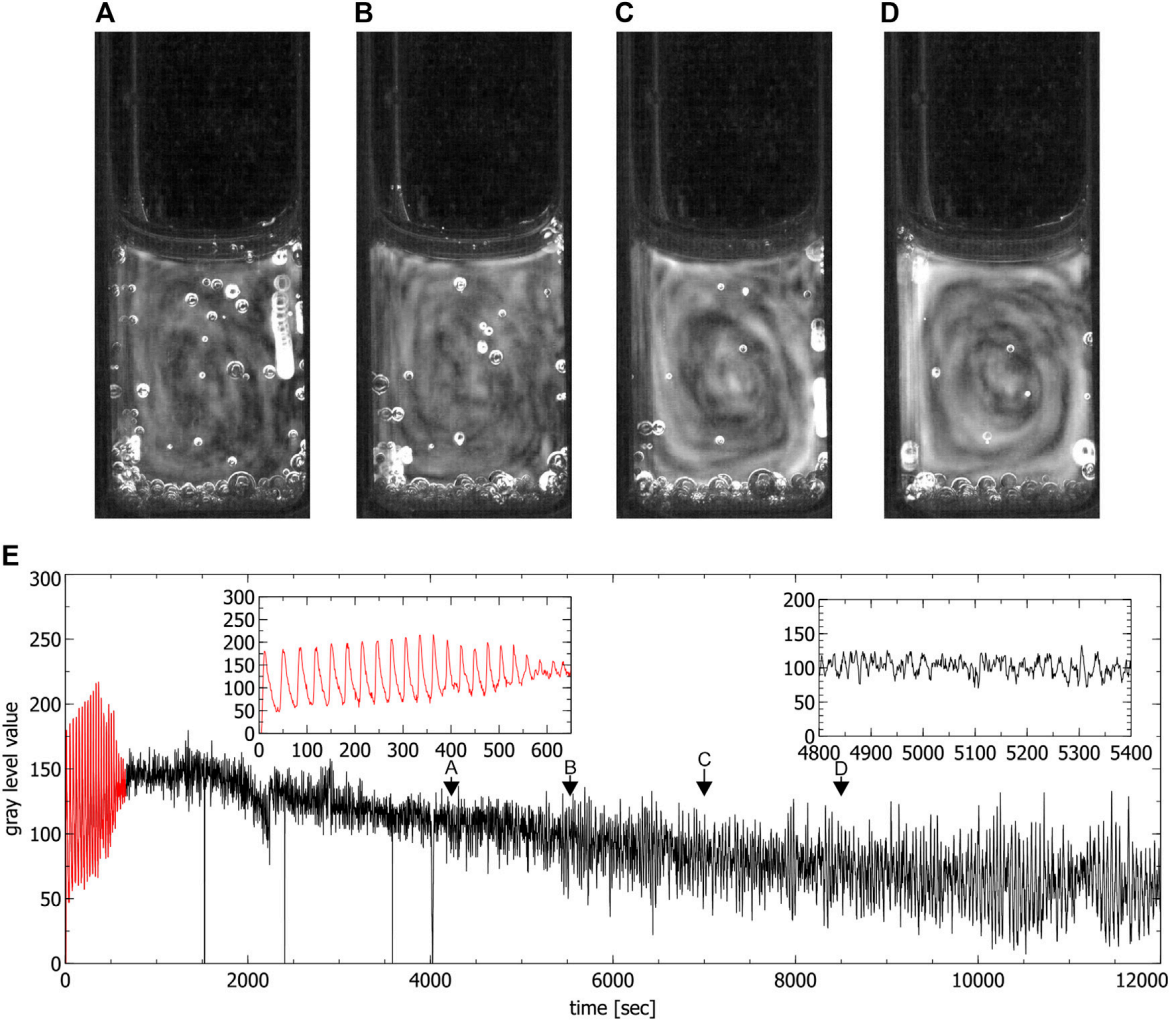
Convection cells in a partly filled standard cuvette during the chaotic phase. **(A–D)** show the presence of one convection cell (liquid moves anti-clockwise). Images were obtained by averaging over 60 s, **(E)** is the time series extracted from the video data where the red part corresponds to the periodic phase. Insets show the periodic phase (left) and part of the chaotic phase (right) magnified (spikes in the time series correspond to the passage of bubbles). Arrows indicate times where the images **(A–D)** were created.

In the fully filled standard cuvette only up- and downwards convective motions were observed (see Figure 5). The fact that in this case no clear convection cells were visible seems to be connected to the ratio between the height and the width of the reaction solution which in this case did not favor the formation of convection cells. By only partially filling the cuvettes, the reaction volume is reduced, this ratio changes and the formation of one single convection cell could be observed which confirms this assumption (see in Figure 6).

**FIGURE 7:**
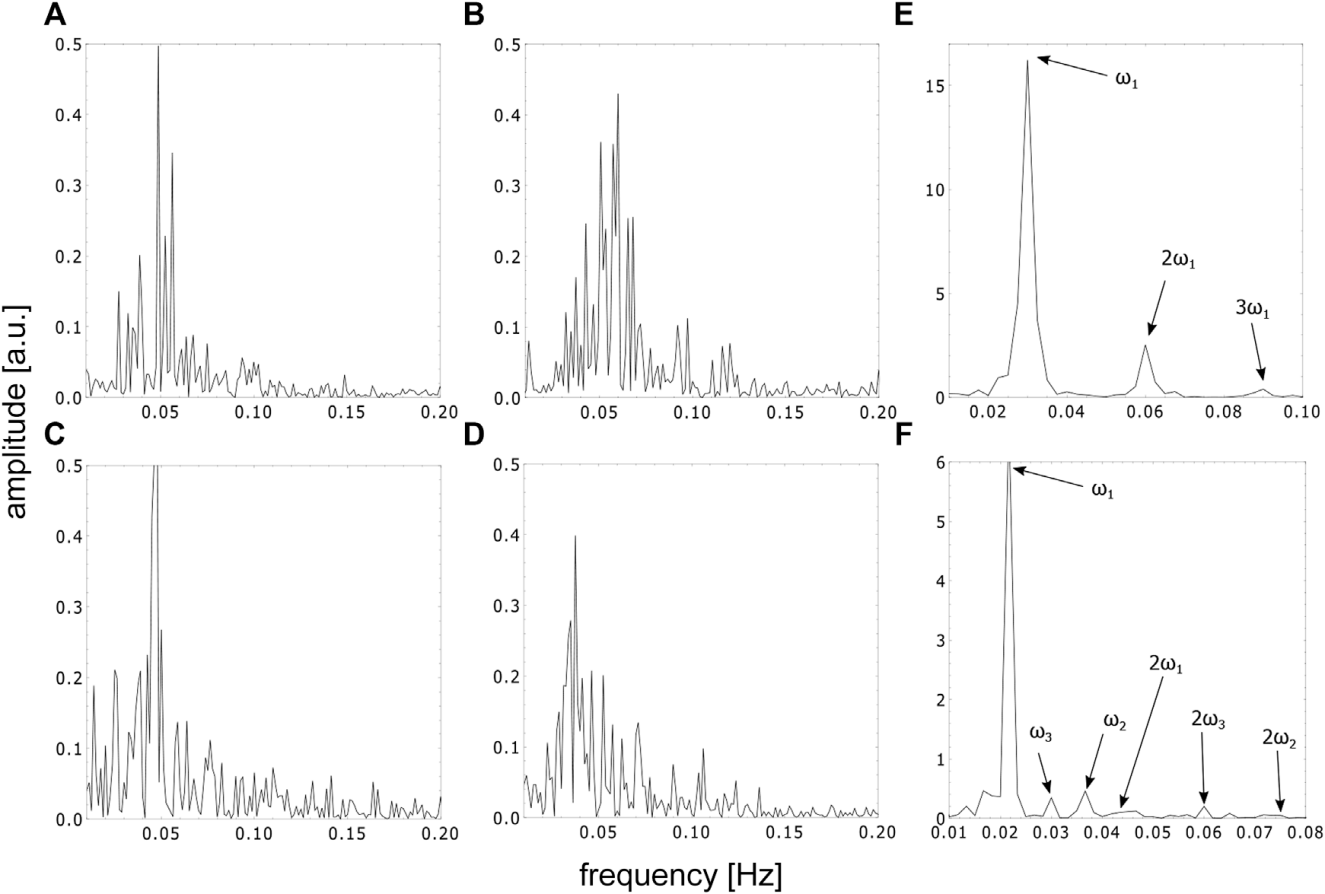
FFT spectra of the time series in the special cuvette in different phases, **(A–D)** represent the FFT spectra of the time series in the aperiodic phase corresponding to the timeframes shown in Figures 4A–D, **(E)** represents the FFT spectra of the first periodic and **(F)** represents the beginning of the second periodic phase, respectively.

The appearance of ordered spatial structures in the chaotic phase in the special cuvette was unexpected. A Fast Fourier Transform (FFT) analysis and the calculation of the maximal Lyapunov exponents of the corresponding aperiodic time series of this cuvette indicates the presence of deterministic chaos. The analysis was conducted in the timeframes indicated by the arrows in Figure 4E. As expected, a broad band spectrum was found (see Figures 7A–D) and the corresponding maximal Lyapunov exponents were all positive (*λ*_*A*_ = 0.001178 ± 0.000276, *λ*_*B*_ = 0.005099 ± 0.000249, *λ*_*C*_ = 0.0009 ± 0.000271 and *λ*_*D*_ = 0.001432 ± 0.000266). This confirms indeed the chaoticity of this phase from a local chemical point of view. Nevertheless, from a global hydrodynamical point of view, the phase is spatially ordered.

For comparison, a Fast Fourier Transform (FFT) analysis was also conducted on the periodic phases of the time series in the special cuvette for the first periodic phase (see Figure 7E) and for the beginning of the second periodic phase (see Figure 7F). The FFT spectrum of the first periodic phase shows a characteristic main frequency and its harmonics. The FFT spectrum of the beginning of the second periodic phase, on the other hand, shows three incommensurable frequencies and its harmonics, indicating the transition from chaos back to periodicity.

Convection cells can form, for different reasons, in liquid and gaseous phases and their form and dynamics depend, respectively, on the properties of the liquid or gas. The fact that in our case the cuvettes could not be closed allows the evaporation of the reaction solution. Subsequently surface tension gradients are created, potentially inducing convections in the bulk phase according to a thermo-capillary Marangoni effect. Even though such an effect becomes weaker with the depths of the liquid layer (Rongy and De Wit, 2007; Rossi et al., 2012), it could play a role in our system. On the other hand, the different densities of the reaction products within the reaction solution could create density gradients big enough to trigger Rayleigh-Bénard convections. Most likely the convection cells observed in our system are an interplay of both effects, where the partially evaporation of the solvent induces a convective motion, which enhances and amplifies already existing Rayleigh-Bénard convections induced by density gradient of the reaction products. This idea is also supported by the appearance of similar convection cells in the work of Gentili et al. (2014). Nevertheless, to get a better picture on the origin of the convection cells in our system, further research is needed.

The fact that chemical chaos and convective motion are connected in the unstirred BZ reaction was already mentioned in the introduction and is confirmed by experiments and theoretical models (Masia et al., 2001; Marchettini et al., 2010; Budroni et al., 2017). The possibility of the coexistence of chemical chaos and hydrodynamical order in form of ordered convective motion is surprising and needs further analysis and modelling.

A first explanation of this coexistence could come from the decoupling of reaction kinetics, diffusion and convection within an unstirred BZ reaction, i.e. the change in relative importance of reaction kinetics, diffusion and convection throughout the evolution of the system (Marchettini et al., 2010).

It is possible to imagine that an increase in importance of convective motion could lead to a “de-synchronization” of the locally in-phase oscillations of the bulk phase. In other words, the relative increase of convection might lead to a mixing of the bulk solution and could therefore increase already existing small gradients in the concentration of the reactants of the system, leading to small “patches” in the solution that change color at different times in an asynchronous way. This relative increase of hydrodynamical effects over chemical dynamics in the chaotic phase is also confirmed in our experiments by the fact that the observed magnitude of the convection velocity is about 30 times faster than the velocity by which the color changes. This is also the reason why the convections becomes visible to the eye. The transition back to periodicity occurs when the hydrodynamical effects become again less important in respect to the chemical kinetics and diffusion.

Apart from the observations described above, we also discovered an interesting phenomenon. There are indications that the chaotic phase lasts longer when clear convection cells are visible. This would mean that the transition back to periodicity is accompanied by the decrease of hydrodynamical order.

To better understand the complex dynamics happening in the system, we investigated the problem also theoretically. By combining the classical equation for reaction and diffusion with the Navier-Stokes equation in a Boussinesq approximation, we developed a simple model. Within this model it was possible to qualitatively confirm the experimental observed behavior.

## Theoretical Model

In order to describe mathematically the above presented phenomena, we took into account the main processes which occur in the reaction cuvette. Firstly, we must consider the chemical reactions leading to localized inhomogeneities of the concentrations and the density. As a consequence, two other effects take place, namely the diffusion and convection of the liquid. In this model we neglect both the surface tension and the evaporation at the free upper surface of the liquid. The three processes taken into consideration are described by combining the classical differential equations for diffusion and reaction kinetics and the Navier-Stokes equation in a Boussinesq approximation (Glansdorf and Prigogine, 1971; Landau and Lifshitz, 2007) which are given by.

$$\frac{\partial c_{i}}{\partial t}+\left(\vec{u}\;\nabla \right)c_{i}=D_{i}\;\Delta c_{i}+f_{i}\left(c\right),$$ (1a)

$$\frac{\partial \vec{\Omega }}{\partial t}+\left(\vec{u}\;\nabla \right)\vec{\Omega }=\nu \Delta \vec{\Omega }+\left(\vec{\Omega }\;\nabla \right)\vec{u}-\vec{\Omega }\left(\nabla \;\vec{u}\right)-\nabla \times \left(\frac{\nabla P}{\rho }\right).$$ (1b)

Here, *c*_*i*_ are the concentrations of the representative reactants, *c*_1_ = [*HBrO*_2_], *c*_2_ = [*Fe* ^3+^] and $c_{3}=\left[BrO_{3}^{-}\right]$ , with the corresponding chemical reactions rates *f*_*i*_(*c*) which are given by.

$$f_{1}\left(c_{1},c_{2},c_{3}\right)=\frac{1}{\epsilon }\left(\frac{qc_{3}-c_{1}}{qc_{3}+c_{1}}f\;c_{2}+c_{1}c_{3}-c_{1}^{2}\right),$$ (2a)

$$f_{2}\left(c_{1},c_{2},c_{3}\right)=c_{3}\;c_{1}-\;c_{2},$$ (2b)

$$f_{3}\left(c_{1},c_{2},c_{3}\right)=c_{1}\left(\frac{1}{2}c_{1}-c_{3}\right)-\frac{q\;f\;\;c_{2}\;c_{3}}{c_{1}+qc_{3}}.$$ (2c)

Eq. 2 are derived from the adapted Oregonator equations (Marchettini et al., 2010) where *ϵ*, *q* and *f* are kinetic parameters. Furthermore, $\overset{⃗}{u}$ and $\vec{\Omega }=\nabla \times \vec{u}$ represent, respectively, the fluid velocity and its vorticity, whereas *P* and *ρ* are, respectively, the pressure and the density of the bulk. Because of the incompressibility of the bulk, the term $\nabla \vec{u}$ vanishes in Eq. 1b. The estimation of the Reynolds number, with respect to the whole cuvette, is about 5.52. Although this value is bigger than 1, it is much smaller than the typical values (of the order of 10^7^). Consequently, in a first approximation it is reasonable to neglect the nonlinear term in $\overset{⃗}{u}$ in the Navier-Stokes Eq. 1b in order to simplify the equation. Furthermore the Boussinesq approximation (adapted for the concentration instead of the temperature (Marchettini et al., 2010)) was applied and a linear analysis around the steady state was performed (for details, see Supplementary Material S1). We obtained an expression for the velocity field for the lowest modes given by

$$\left(u_{2},u_{3}\right)=\pi \chi \left(\frac{1}{b}\sin\left(\frac{2\pi \xi_{2}}{a}\right)\cos\left(\frac{\pi \xi_{3}}{b}\right),-\frac{2}{a}\cos\left(\frac{2\pi \xi_{2}}{a}\right)\sin\left(\frac{\pi \xi_{3}}{b}\right)\right)$$ (3)

where *u*_2_ and *u*_3_, *ξ*_2_ and *ξ*_3_ are the dimensionless y- and z-component of the velocity field as well as the dimensionless y- and the z-coordinate, respectively. *a* and *b* in this formula represent the dimensions of the liquid in the cuvette. The function *χ* is independent of the dimensionless coordinates. We also obtained an algebraic relation between reaction related parameters and those related to the hydrodynamics given by

$$\frac{c_{3}^{*}}{\epsilon }=\pi^{2}\frac{1+\delta_{2}\pi^{2}\left(\frac{4n^{2}}{a^{2}}+\frac{m^{2}}{b^{2}}\right)}{1+f+\delta_{2}\pi^{2}\left(\frac{4n^{2}}{a^{2}}+\frac{m^{2}}{b^{2}}\right)}\left(\frac{4n^{2}}{a^{2}}+\frac{m^{2}}{b^{2}}\right),$$ (4)

where $c_{3}^{*}$ and *δ*_2_ are, respectively, the steady state approximation of the concentration of $\left[BrO_{3}^{-}\right]$ and the dimensionless diffusion coefficient of ferriin. *n* and *m* are the modes of the harmonic functions of the velocity field.

The foregoing expression enables us to qualitatively compare the hydrodynamic chaos with the chemical order. The smaller the numbers *n* and *m* are, the clearer is the structure of the convection pattern and the more ordered is the flow. The variation interval of $c_{3}^{*}$ is strongly affected by formula (4). The value of $c_{3}^{*}$ can be indirectly calculated relating to the original Oregonator model and gives the range

$$c_{3}^{*}\in \left(2\times 10^{-4},1.0355\right)$$ (5)

for the appearance of oscillations (using *f* ∈ (0.5, 2.4) as found by Scott (Scott, 1993)). The threshold value found by Marchettini et al. (Marchettini et al., 2010) for the concentration $\bar{c_{3}}$ is $\bar{c_{3}}\approx 0.95$ and thus is comparable to what we found in this study (for details, see Supplementary Material S1).

**FIGURE 8:**
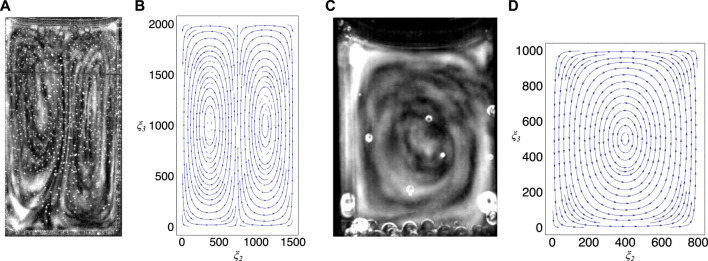
Comparison between experiment and theory, **(A)** shows the convection cells developed in a special cuvette in the chaotic phase (see also Figure 2A), **(B)** shows a stream plot that indicates the field lines of the velocity field given in formula (3) by using the values given in the text, **(C)** shows the convection cells developed in a partly filled standard cuvette (see also Figure 6D), **(D)** shows a stream plot that indicates the field lines of the velocity field given by the three-dimensional extension of the model (for details, see text).

In our case, for *n* = *m* = 1 (hydrodynamic order) and for *a* = 1500, *b* = 2000 and *δ*_2_ = 0.558, $c_{3}^{*}\in \left(6\times 10^{-8},1.23\times 10^{-7}\right)$ , thus outside of the chemical order. By increasing the values of *n* and *m*, i.e. increasing the hydrodynamic disorder, the value of $c_{3}^{*}$ moves within the range of chemical order. For *n* = 100 and *m* = 1 this is already the case, $c_{3}^{*}\in \left(5.6\times 10^{-4},1.22\times 10^{-3}\right)$ . These results prompt qualitatively that the hydrodynamic order is indeed connected to chemical chaos, as seen from the experimental results. Figure 8 compares the real dynamics in the special cuvette with the velocity field calculated according to the obtained formula (3) using the above initially mentioned values for *n*, *m*, *a*, *b* and *δ*_2_. We stress the fact that formula (3) and its implications occur only within a 2-dimensional model, applicable only for the slim special cuvette.

To model the dynamics in the standard cuvette we extended the procedure to the three-dimensional case. This case is partially different from the two-dimensional one, even though the results are only slightly different from formula 3 and formula 4. In this three-dimensional case a single convection cell, as observed experimentally, may be obtained using the lowest modes in the slightly different equation for the velocity field (Figure 8D shows the velocity field for this case by using the dimensions *a* = 800 and *b* = 1000 that correspond to a partly filled standard cuvette) (for details, see Supplementary Material S1). Within this simple model the number of convection cells depend only on the boundary conditions chosen and not on the ratio between the width and the height of the cuvette as one would expect from a more sophisticated model.

## Conclusion

The behavior of an unstirred BZ reaction in a batch configuration is more complex than it is in constantly stirred tank reactors or in Petri dishes. This is due to the complex coupling between reaction kinetics, convection and diffusion. In both cases either convection and diffusion or convection only is suppressed. Normally, a BZ reaction in a batch configuration is investigated in a spectrophotometer, where possible inhomogeneities or convective motions in the bulk solution cannot be seen.

In this work we focused on these inhomogeneities and we investigated the convective motion created in the chaotic phase in an unstirred BZ reaction in a cuvette configuration. The ordered convective cells observed in specific cuvette geometries were investigated in respect to the local chemical kinetics in the reaction solution. Surprisingly, we discovered that this ordered hydrodynamical structures are correlated to the chaotic nature of the local chemical kinetics. As a first explanation of this correlation, we assume that the formed convection cells lead to a “de-synchronization” of the previously homogeneous color oscillations moving the system into a chaotic phase. With the simple mathematical model presented here we were able to indirectly confirm the same qualitative results, i.e. that a connection between chemical chaos and hydrodynamical order in such systems exists.

## Data Availability

The original contributions presented in the study are included in the article/Supplementary Material, further inquiries can be directed to the corresponding author.

## Supplementary Material

Supplementary Material S1 Details on the data treatment and analysis and the theoretical model used in the main article.

Supplementary Material S2 Video of the full dynamics of an unstirred ferroin-catalyzed BZ reaction in a special cuvette (200x faster than real time).
