# Supplementary material for: Periodic Motion in the Chaotic Phase of an Unstirred Ferroin-Catalyzed Belousov Zhabotinsky Reaction
Source: Front Chem. 2022 Jul 8;10:881691. doi: 10.3389/fchem.2022.881691 (PMC9304747; doi:10.3389/fchem.2022.881691)
Supplement: Supplementary file 1 [file DataSheet1.pdf]

Supplementary Material

1 OPTICAL SETUP

To follow the reaction dynamics a simple optical setup was used consisting of a ceiling LED panel (B.K.Licht 18 Watt LED Panel, 2400 lm, 4000K white light) as background light and a standard video camera (JVC Everio HM400) for data aquisition which was recorded on a PC (details see Fig. S1, **(a)**).

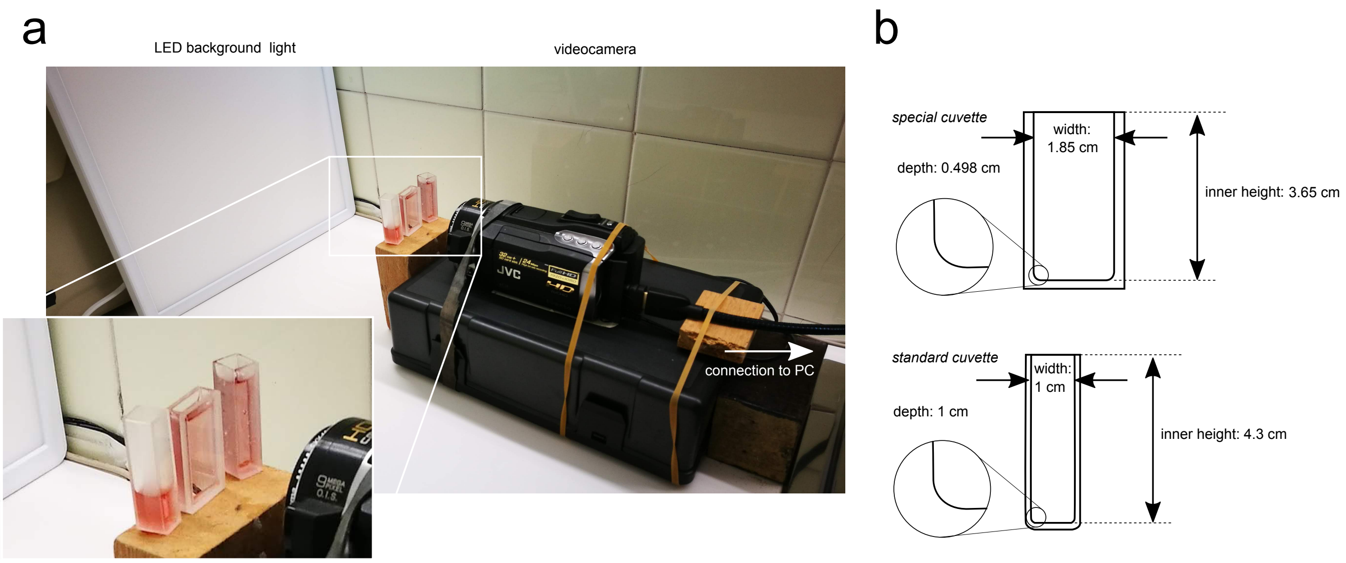

Figure S1. Optical setup, **(a)** shows arrangement of the LED backlight, spectrophotometric quartz cuvettes and the video camera; inset shows a zoom on the cuvettes, **(b)** shows the schemes of the used cuvettes with their dimensions; insets show the curved inside corners.

Three cuvettes (two standard, one special cuvette (middle)) were put in the focal point of the video camera. The two standard cuvettes on each corner where inclinded slightly to adjust to the optical distortion of the video camera. The camera itself was placed on a solid support fixed with rubber bands. The dimensions of the used cuvettes are shown in Fig. S1, **(b)**.

2 DATA TREATMENT

The video signal was recorded with the video software VLC media player with an aquisition rate of 0.5 to 1 images per second at high definition (HD) (1280 x 720 pixels). The images then were treated with the image processing package Fiji (imageJ2).
The video camera JVC Everio HM400 uses a CMOS-Chip with Bayer RGB Color Filter Array. The blue filter in this array has a sensitivity peak at around 450 nm [9]. Ferriin, the oxidized blue form of the color indicator has a minimum at 450 nm [10]. This is the reason why we selected the blue channel of the video signal to analyze the dynamics of the reaction system. The information of a single channel is coded in 8-bit (256) different gray values from 0 (white) to 255 (black). High gray level values therefore correspond to low concentration of ferriin.

2.1 Extraction of the Time Series from the Video Data

To extract the time series from an video signal the video is transformed into a series of images. As mentioned above, the images were treated with the image processing package Fiji (imageJ2). This package is able to create a *"Kymograph"* (see Fig. S2 (b)), i.e. a space-time plot of the pixel values of a selected cross-section line (see Fig. S2 (a) green line) throughout an image series.
In this way we have transformed the entire video into an image that contains the whole dynamics of the system at a certain line. In our case, it illustrates clearly the periodicity of the color change in the beginning and the gradually transition to chaos.

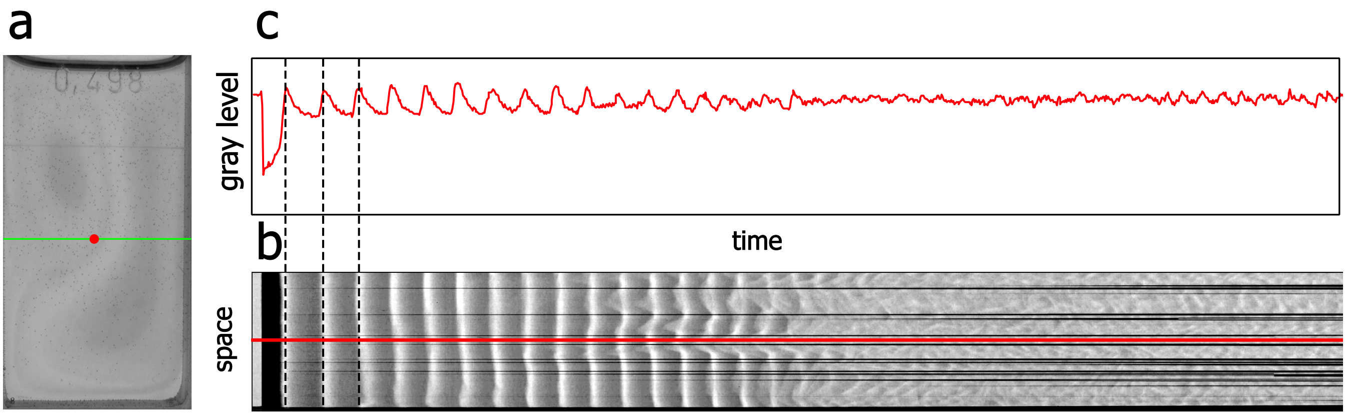

Figure S2. Extraction of the time series from video data, **(a)** shows an image of the cuvette filled with the reaction solution, **(b)** shows a space-time plot of the full image series showing the periodic phase and the transition to the chaotic one, **(c)** shows the time series of the gray level values at the red point in (a).

Since we are interested in the dynamics of a given point (see Fig. S2 (a) red point) which corresponds to a line in the Kymograph (see Fig. S2 (b) red line) we translate the shades of gray back into gray level values by performing a *"Profile Plot"* and obtain Fig. S2 (c).

The time series extracted from the video data in the above described way is comparable to a time series obtained by a spectrophotometer at a wavelength of 450 nm. The advantage of an image series of the system over a spectrophotometric time series is that a time series can be in principle extracted at any point in the solution.

2.2 Visualization of Convection Cells

To visualize the convective motion in one image only, a time average over 60 seconds was calculated. The images were treated with the image processing package Fiji (imageJ2) where the option *"image > stack > Z project"* was used. Here as *"Projection type" "Standard deviation"* was used. In supplementary material S2 a video of the full reaction dynamics is shown (200x faster than real time).

2.3 Calculation of the Lyapunov Exponents

To ensure that the so called "chaotic" phase in our time series shows indeed typical properties of chaotic behavior we analyzed the corresponding time series thoroughly.
A typical property of chaotic behavior is a broad band spectra in the frequency domain of the corresponding aperiodic oscillations as shown in the main article in Fig. 7 A-D. Another typical property of chaotic

behavior is a positive maximal Lyapunov exponent. Rossi et al. have already shown that this is the case in the phase of aperiodic oscillations in ferroin-catalyzed unstirred BZ reactions [6].
To show that the chaotic phases in the time series obtained from the video data of our experiments shows also this property we calculated the maximal Lyapunov exponent. For this procedure the TISEAN Nonlinear Time Series Analysis package [3] was used. For calculating the Lyapunov exponent of the chaotic phase in the experiment shown in Fig. 1 of the main article (an experiment conducted in a fully filled standard cuvette), the first 1000 seconds of the chaotic phase (1400-2400s) were used. Fig S3 shows the calculated local correlation entropy $S(\varepsilon, n, m)$ as a function of the discrete time steps n, the embedding dimension M (encoded in the shades of the different colors; pale shade $M = 2$, darker shade $M = 3$ and darkest shade $M = 4$; for small ε values only $M = 2$ and $M = 3$ is shown) and the initial neighborhood size ε. The initial slope of the local correlation entropy gives the maximum Lyapunov exponent.

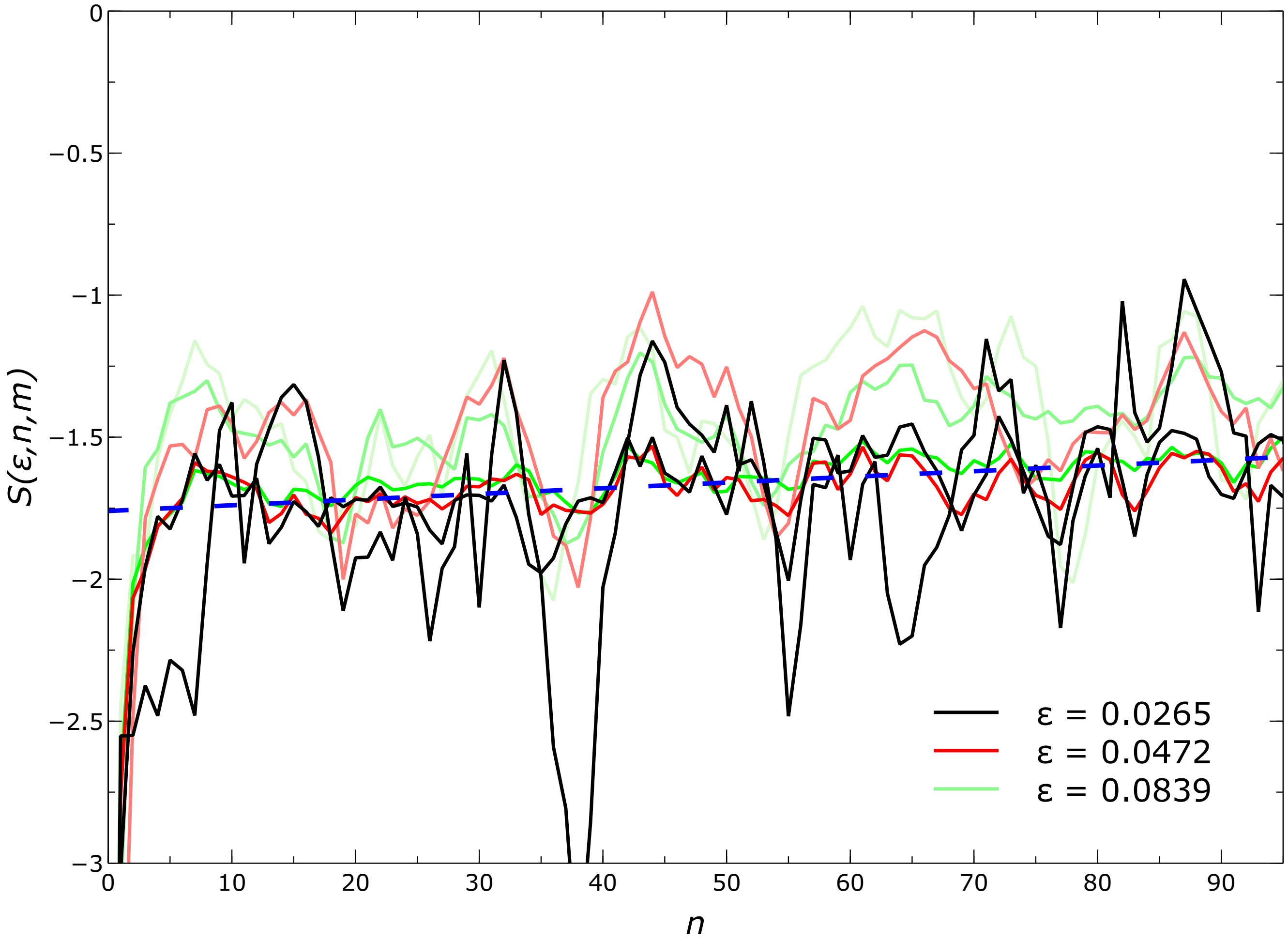

Figure S3. The local correlation entropy $S(\varepsilon, n, m)$ as a function of the initial neighborhood size ε, the discrete time steps n and the embedding dimension M (for details see text)

The fitted slope (blue dashed line in Fig. S3) for n in the range of (5,90) gives a value of 0.00256187 ± 0.0003971. This value is comparable to 0.00532 ± 0.00002 which is the value found by Rossi et al. for the chaotic phase in their experiment.

The above described procedure was repeated for the time points marked in the chaotic phase in the experiment that was conducted in the special cuvette (see Fig. 4 of the main article). All values for the maximum Lyapunov exponent were positive ($\lambda_A = 0.001178 \pm 0.000276$, $\lambda_B = 0.005099 \pm 0.000249$, $\lambda_C = 0.0009 \pm 0.000271$ and $\lambda_D = 0.001432 \pm 0.000266$). This confirms indeed the chaoticity of the chaotic phase.

3 THEORETICAL MODEL

In order to describe mathematically the above presented phenomena, we took into account the main processes which occur in the reaction cuvette. Firstly, we must consider the chemical reactions leading to localized inhomogeneities of the concentrations and the density. As a consequence, two other effects take place, namely the diffusion and convection of the liquid. In this model we neglect both the surface tension and the evaporation at the free upper surface of the liquid. The three processes taken into consideration are described by combining the classical differential equations for diffusion and reaction kinetics and the Navier-Stokes equation [4, 2] which are given by:

$$\frac{\partial c_i}{\partial t} + (\vec{u}\,\nabla)c_i = D_i\,\Delta c_i + f_i(c)\,, \tag{S1a}$$

$$\frac{\partial \vec{\Omega}}{\partial t} + (\vec{u}\,\nabla)\vec{\Omega} = \nu\Delta\vec{\Omega} + (\vec{\Omega}\,\nabla)\vec{u} - \vec{\Omega}(\nabla\,\vec{u}) - \nabla\times\Big(\frac{\nabla P}{\rho}\Big)\,. \tag{S1b}$$

Here c_i are the concentrations of the representative reactants, $c_1 = [HBrO_2]$, $c_2 = [Fe^{3+}]$ and $c_3 = [BrO_3^-]$. The corresponding chemical reactions rates $f_i(c)$ are given by

$$f_1(c_1, c_2, c_3) = \frac{1}{\epsilon}\Big(\frac{qc_3 - c_1}{qc_3 + c_1} f\, c_2 + c_1 c_3 - c_1^2\Big)\,, \tag{S2a}$$

$$f_2(c_1, c_2, c_3) = c_3\, c_1 - \, c_2\,, \tag{S2b}$$

$$f_3(c_1, c_2, c_3) = c_1\Big(\frac{1}{2}c_1 - c_3\Big) - \frac{q\,f\;c_2\,c_3}{c_1 + qc_3}\,. \tag{S2c}$$

Furthermore, $\vec{u}$ and $\vec{\Omega} = \nabla\times\vec{u}$ represent, respectively, the fluid velocity and its vorticity, whereas *P* and ρ are the pressure and the density of the bulk, respectively. Because of the incompressibility of the bulk, the term $\nabla\vec{u}$ vanishes in equation S1b. The estimation of the Reynolds number, with respect to the whole cuvette, is about 5.52. Although this value is bigger than one, it is much smaller than typical values (of the order of 10^7). Consequently it is reasonable to neglect the nonlinear term in $\vec{u}$ in the Navier - Stokes equation (S1b) simplifies to

$$\frac{\partial \vec{\Omega}}{\partial t} = \nu\vec{\Omega} - \nabla\times\Big(\frac{\nabla P}{\rho}\Big) \tag{S3}$$

Any way the solution obtained in formula (S24) leads to automatic disappearance of the nonlinear terms. Now, applying the Boussinesq approximation (adapted for the concentration instead of the temperature [5]), the concentrations slightly deviate from the homogeneous average and can be written as

$$c_i = \overline{c}_i + c_i^{'} \tag{S4}$$

where $\overline{c}_i$ denotes the average value of the concentrations and $c_i^{'}$ its deviations. Even if $\overline{c}_i$ would be time dependent, we would have

$$\nabla c_i = \nabla c_i^{'} \tag{S5}$$

Also, because of the presence of these concentrations deviations, the bulk density may have small inhomogeneities and the pressure may deviate from its equilibrium value. Denoting with $\overline{\rho}$ the average density and with P_0 the pressure at the equilibrium, we have

$$\begin{aligned} \rho &= \overline{\rho} + \sum_{i=1}^{3} \left.\frac{\partial \rho}{\partial c_i}\right|_{\overline{c}} c_i^{'} \,, \\ P &= P_0 + P^{'} = -\rho g z + P^{'} \,. \end{aligned} \tag{S6}$$

By keeping only the first order terms of the perturbing prime quantities, Eqs.(S1a) and (S1b) become

$$\frac{\partial c_i}{\partial t} + (\vec{u}\,\nabla) c_i^{'} = D_i \Delta c_i + f_i(\overline{c} + c^{'}) \,, \tag{S7a}$$

$$\frac{\partial \vec{\Omega}}{\partial t} = \nu \Delta \vec{\Omega} + \frac{1}{\overline{\rho}} \sum_{i=1}^{3} \left.\frac{\partial \rho}{\partial c_i}\right|_{\overline{c}} \nabla c_i^{'} \times \vec{g} \,. \tag{S7b}$$

Here, we are interested in performing a linear analysis around the steady state of equation (S7) instead of its full mathematical solution. Being at the steady state means that $\partial_t c_i = 0$ and $\partial_t \vec{\Omega} = 0$. Also, thanks to the Boussinesq approximation, we have $f_i(\overline{c}) = 0$ (remember that $\overline{c}_i$ are homogeneous). Consequently, we obtain

$$\overline{c} = (0, 0, c_3^*) \tag{S8}$$

where c_3^* remains an undetermined, free parameter. The chemical rates f_i may be developed around $\overline{c}$ as follows

$$f_i(\overline{c} + c^{'}) \approx \sum_{i=1}^{3} \left.\frac{\partial f_i}{\partial c_j}\right|_{\overline{c}} c_j^{'} \tag{S9}$$

Substituting in system (S7) and indicating with a star the quantities referring to the steady state, we get

$$(\vec{u}^* \nabla)c_i^{'} = D_i \Delta c_i^{'} + \sum_{i=1}^{3} \frac{\partial f_i}{\partial c_j}\bigg|_{\overline{c}} c_j^{'} \,, \tag{S10a}$$

$$\nu \Delta \vec{\Omega}^* + \frac{1}{\rho} \sum_{i=1}^{3} M_i \bigg|_{\overline{c}} \nabla c_i^{'} \times \vec{g} = 0 \,. \tag{S10b}$$

where M_i express the molar masses of the chemical species. To continue the calculations, we refer to the geometrical system depicted in Fig. S4.

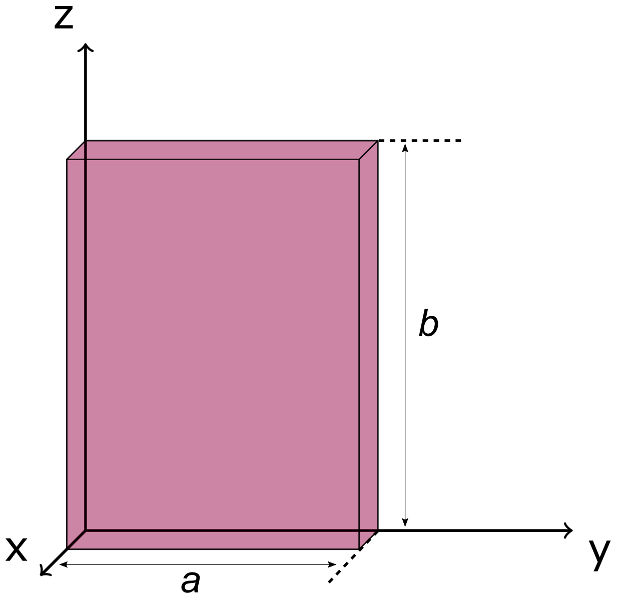

Figure S4. Vertical slab representing the cuvette used in the model. *a* and *b* being the dimensions of the liquid.

3.1 Two-dimensional Approximation

Since the width can be considered negligeable compared to the other two dimensions, we will consider that c_1, c_2, c_3 and $\vec{u}$ depend just on (y, z). Then, the dimensionless version of the system (S10) gets the form

$$(\vec{u}_\tau^* \nabla)c_i^{'} = \delta_i \Delta c_i^{'} + \sum_{i=1}^{3} a_{ij} c_j^{'} \,, \tag{S11a}$$

$$\Delta \omega_1^* - \sum_{i=1}^{3} |Ra_i| \frac{\partial c_i^{'}}{\partial \xi_2} = 0 \,. \tag{S11b}$$

where $\vec{u}_\tau^* = (u_2^*, u_3^*)$ is the steady state velocity expressed in the dimensionless coordinates $\vec{\xi} = (\xi_2, \xi_3)$, a_{ij} are the dimensionless variante of $\partial f_i / \partial c_j|_{\overline{c}}$ and ω_1^* is the single non-vanishing component of the dimensionless vorticity $\vec{\Omega}$. The numbers Ra_i represent the Rayleigh-numbers given by [5]:

$$Ra_i = -\frac{gL_0^3}{\overline{\rho} D_i \nu} \frac{\partial \rho}{\partial c_i}\bigg|_{\overline{c}} \tag{S12}$$

where g, L_0, ρ, D_i and ν are, respectively, the gravitational acceleration, the length gauge, the density, the diffusion coefficient and the kinematic viscosity. Four our system we have

$$Ra_1 = -0.37\,, \qquad Ra_2 = -758\,, \qquad Ra_3 = -41.5 \tag{S13}$$

As the hydrodynamical model is reduced to two dimensions, it is sufficient to consider only the stream function Ψ, related to the velocities in the following manner

$$u_2 = \frac{\partial \Psi}{\partial \xi_3}\,, \qquad u_3 = -\frac{\partial \Psi}{\partial \xi_2} \tag{S14}$$

The four unknown functions c_1, c_2, c_3 and Ψ are related by the following equations (hereafter we write c_i for $c_i^{'}$ and u for u^*)

$$\Big(\frac{\partial \Psi}{\partial \xi_3}\frac{\partial}{\partial \xi_2} - \frac{\partial \Psi}{\partial \xi_2}\frac{\partial}{\partial \xi_3}\Big) c_i = \delta_i \Delta c_i + f_i(c)\,, \tag{S15a}$$

$$\Delta^2 \Psi - \sum_{i=1}^{3} Ra_i \frac{\partial c_i}{\partial \xi_2} = 0\,. \tag{S15b}$$

It is interesting to note that $\omega_1 = -\Delta\Psi$. The Laplace operator $\nabla^2\Psi$ is calculated only with respect to ξ_2 and ξ_3. As it is known, the reaction rates $f_i(c)$ [see equation (S2)] are dimensionless quantities, so the involved constants are pure numbers

$$\epsilon = 10^{-2}\,, \quad q = 9.5 \times 10^{-5}\,, \quad \delta_1 = 1\,, \quad \delta_2 = 0.558\,, \quad \delta_3 = 0.959\,. \tag{S16}$$

The stoichiometric coefficient $f \in (0.495,\ 2.4)$ for the periodic chemical regime [8]. The initial concentrations are homogeneous and have the following values

$$c_1(0) = fq\,, \quad c_2(0) = 2.7 \times 10^{-5}\,, \quad c_3(0) = 0.0156 \tag{S17}$$

The complete formal solution of the system (S15) will be addressed to a forthcoming publication. As mentioned above, in this article we intend to give qualitative hints explaining the onset of the convection cells and their relation to the periodic (or chaotic) chemical regime. The pattern of the convection cells is given by the velocity field (u_2, u_3). Control parameters of the chaotic/periodic chemical regime may be obtained by the steady state values of c_3^* and the free stoichiometric coefficient f. Let us consider the velocities $\vec{u}_\tau = (\partial_{\xi_3}\Psi, -\partial_{\xi_2}\Psi)$ in equation (S15) as free unknown parametric functions and make the Ansatz

$$c_i = c_{0i}\, e^{\vec{\kappa}\cdot\vec{\xi}} \tag{S18}$$

in equation (S15a). This implies a secular equation for the paramater $\vec{\kappa}\cdot\vec{u}_\tau$

$$\left(\vec{k}\cdot\vec{u}_\tau - \delta_1\kappa^2 - \frac{c_3^*}{\epsilon}\right)\left(\vec{k}\cdot\vec{u}_\tau + 1 - \delta_2\kappa^2\right) - \frac{c_3^* f}{\epsilon} = 0 \tag{S19}$$

having the solution

$$\begin{aligned}(\vec{k}\cdot\vec{u}_\tau)_{1,2} = \frac{1}{2}\Bigg\{ \pm \sqrt{\left[(\delta_1+\delta_2)\kappa^2 + \frac{c_3^*}{\epsilon} - 1\right]^2 + 4\left[\left(\delta_1\kappa^2 + \frac{c_3^*}{\epsilon}\right)(1-\delta_2\kappa^2) + \frac{c_3^*}{\epsilon}\right]} + \\ + (\delta_1+\delta_2)\kappa^2 + \frac{c_3^*}{\epsilon} - 1\Bigg\}\end{aligned} \tag{S20}$$

Though $\vec{u}_\tau$ is a given function on $\vec{\xi}$, the r.h.s. of the foregoing expression shows us that $\vec{k}\cdot\vec{u}_\tau$ is independent of $\vec{\xi}$. As a consequence, the term $\vec{k}\cdot\vec{u}_\tau$ can be considered as a number representing a constraint between the coordinates of the flow velocities. Depending on $\vec{k}\cdot\vec{u}_\tau$, we can express the amplitudes of the deviations of the concentrations from the homogeneous average in the steady state

$$c_{02} = \frac{c_3^*\, c_{01}}{1 + \vec{k}\cdot\vec{u}_\tau - \delta_2\kappa^2}\,, \tag{S21a}$$

$$c_{03} = \left(1 + \frac{f}{1 + \vec{k}\cdot\vec{u}_\tau - \delta_2\kappa^2}\right)\frac{c_{01}c_3^*}{\delta_3\kappa^2 - \vec{k}\cdot\vec{u}_\tau}\,. \tag{S21b}$$

when c_{01} is still unknown. Substituting the previous result in equation (S15b), we obtain

$$\Delta\omega_1^* = \sum_{i=1}^{3} |Ra_i|\, c_{0i}\, \kappa_2\, e^{\vec{\kappa}\cdot\vec{\xi}}. \tag{S22}$$

Remembering that $\omega_1^* = -\Delta\Psi$, we obtain the equation for the stream function Ψ in the stationary regime

$$\Delta^2\Psi = -\sum_{i=1}^{3} |Ra_i|\, c_{0i}\, \kappa_2\, e^{\vec{\kappa}\cdot\vec{\xi}} \tag{S23}$$

which solution is

$$\Psi(\vec{\xi}) = \chi(\kappa, R)\, e^{\vec{\kappa}\vec{\xi}} \qquad \text{where} \qquad \chi(\kappa, R) = \sum_{i=1}^{3} |Ra_i|\, c_{0i}\, \kappa_2\, \Phi(\kappa, R) \tag{S24}$$

The term $\Phi(\kappa, R)$ corresponds to

$$\Phi(\kappa,R) = -\frac{\pi}{8}R^4 \ln R\Big\{2\sum_{j=0}^{\infty}\frac{j+1}{j!(j+2)!}\Big(-\frac{\kappa R}{2}\Big)^{2j} + \sum_{j=0}^{\infty}\frac{2j+3}{\Gamma(j+3/2)\Gamma(j+7/2)}\Big(-\frac{\kappa R}{2}\Big)^{2j+1} + \\ -\frac{1}{\ln R}\Big[\sum_{j=0}^{\infty}\frac{(j+1)^2}{\Gamma^2(j+1)}\Big(-\frac{\kappa R}{2}\Big)^{2j} + \sum_{j=0}^{\infty}\frac{(j+3/2)^2}{\Gamma^2(j+7/2)}\Big(-\frac{\kappa R}{2}\Big)^{2j+1}\Big]\Big\} \tag{S25}$$

The number *R* is determined by the following integral

$$\int_0^a\int_0^b e^{-\vec{\kappa}\cdot\vec{\eta}}\,|\vec{\eta}|^2\,\ln|\vec{\eta}|\,d\eta_2 d\eta_3 = -\Phi(\kappa,R) \tag{S26}$$

The former expression implies that *R* lies within the interval $[\min(a,b), \sqrt{a^2+b^2}]$.

The vertical walls of the cuvette interact similarly with the liquid, so we may inmpose the following boundary condition

$$\Psi(\xi_2 = 0) = \Psi(\xi_2 = a) \tag{S27}$$

implying a "quantization" of κ_2

$$\kappa_2 = \frac{2\pi i n}{a}\,, \qquad \text{with } n \text{ an odd integer} \tag{S28}$$

The glass bottom of the cuvette and the free liquid surface are by no means physically equivalent. As stated above, we do not consider the superficial tension and the evaporation at the upper free liquid surface. Instead we impose that, at the two boundaries, the function Ψ behave in an "opposite manner"

$$\Psi(\xi_3 = 0) = -\Psi(\xi_3 = b) \tag{S29}$$

This implies also the "quantization" of the second component of κ

$$\kappa_3 = \frac{\pi i m}{b}\,, \qquad \text{with } m \text{ an odd integer} \tag{S30}$$

Of course this view of the boundary conditions is only one among many others given the multitude of other possible constrains. We chose this one because it corresponds to the observed dynamics in the cuvette used in this model. According to Saltzmann [7, 1], we can consider a fraction of all possible modes of $\Psi(\vec{\xi})$

$$\Psi(\vec{\xi}) = \chi(n, m, R) \sin\left(\frac{2\pi n \xi_2}{a}\right) \sin\left(\frac{\pi m \xi_3}{b}\right) \tag{S31}$$

The lowest mode is identified by *n* = *m* = 1. Consequently, we obtain

$$\Psi(\vec{\xi}) = \chi(1, 1, R) \sin\left(\frac{2\pi \xi_2}{a}\right) \sin\left(\frac{\pi \xi_3}{b}\right) \tag{S32}$$

The velocity field is easily obtained

$$(u_2, u_3) = \pi\chi\left(\frac{1}{b}\sin\left(\frac{2\pi\xi_2}{a}\right)\cos\left(\frac{\pi\xi_3}{b}\right), -\frac{2}{a}\cos\left(\frac{2\pi\xi_2}{a}\right)\sin\left(\frac{\pi\xi_3}{b}\right)\right) \tag{S33}$$

This solution corresponds to the observed direction of the flow (see Fig. S5 (b)). Considering the general solution (S24), we can easily verify that

$$\vec{\kappa} \cdot \vec{u}_T = 0 \tag{S34}$$

Substituting this expression in the secular equation (S19), we get a relation between the two control parameters c_3^* and *f*

$$\frac{c_3^*}{\epsilon} = \pi^2 \frac{1 + \delta_2\pi^2(\frac{4n^2}{a^2} + \frac{m^2}{b^2})}{1 + f + \delta_2\pi^2(\frac{4n^2}{a^2} + \frac{m^2}{b^2})}\left(\frac{4n^2}{a^2} + \frac{m^2}{b^2}\right) \tag{S35}$$

The foregoing expression enable us to qualitatively compare the hydrodynamic chaos with the chemical order. The smaller the numbers *n* and *m* are, the clearer is the structure of the convection pattern and the flow is more and more ordered. The variation interval of c_3^* is strongly affected by formula (S35). For fixed *n* and *m*, indeed, the range of c_3^* is determined by the possible variation of the stoichiometric coefficient *f*. Unfortunately, the fixed point expressed by equation (S8) does not admit, at least in the linear analysis, any oscillation around it, within the Marchettini model (S2). It is possible to show that FKN model is a limit case of Marchettini model if we take

$$x := \frac{c_1}{c_3}, \qquad z := \frac{c_2}{c_3^2}, \qquad \epsilon_{FKN} = \frac{\epsilon_1}{c_3} \tag{S36}$$

Scott [8] prooved that the fixpoint x^* in FKN model has instability when *f* lies in the interval

$$\frac{1}{2} + 3q \leqslant f \leqslant (1 + \sqrt{2})\left[1 - (1 + \sqrt{2})q\right] \tag{S37}$$

If we choose

$$x^* = \frac{c_1(0)}{c_3^*} = \frac{fq}{c_3^*} \tag{S38}$$

then we may find the instability range for c_3^*, including oscillations. Combining the formulae (S37) and (S38), we obtain the possible oscillation range c_3^*, which for $q = 9.5 \times 10^{-5} \approx 10^{-4}$ gives

$$c_3^* \in (2 \times 10^{-4}, 1.0355) \tag{S39}$$

We take this interval as a criterium to decide whether our system evolves to the chemical order or not. The threshold found by Marchettini *et al.* [5] is $\bar{c}_3 \approx 0.93$: under this level, the existence of a limit cycle has been noticed and above $\bar{c}_3 \approx 0.95$ the presence of quasi-periodicity and chaos. In our case, for *n* = *m* = 1 (hydrodynamic order) and for our dimensionless cuvette dimensions $a = 1500$ and $b = 2000$ $c_3^* \in (6 \times 10^{-8}, 1.23 \times 10^{-7})$, thus outside of the chemical order. For *n* = 10 and *m* = 1, we are still outside: $c_3^* \in (5.16 \times 10^{-6}, 1.17 \times 10^{-5})$ but for *n* = 100 and *m* = 1 we have $c_3^* \in (5.6 \times 10^{-4}, 1.22 \times 10^{-3})$, thus already within the instability interval. These results prompt qualitatively the hypotheses that the hydrodynamic order is connected to chemical chaos, as seen from the experimental pictures (see Fig. S5).

3.2 Extension to Three Dimensions

The equation (S33) models only the flow dynamics in the special cuvette which is theoretically considered to be two-dimensional. The flow dynamics in the standard cuvette (see e.g. Fig. 5 of the main article) is three-dimensional, therefore a generalization of the model to three dimensions is needed. The corresponding generalization of the system (S11 a,b) has the form:

$$(\vec{u^*}_\tau \nabla) c_i' = \delta_i \Delta {c_i}' + \sum_{i=1}^{3} a_{ij} c_j' \tag{S40 a}$$

$$\Delta \omega_1^* = -\sum_{i=1}^{3} Ra_i \, \frac{\partial c_i'}{\partial \xi_2} \tag{S40 b}$$

$$\Delta \omega_2^* = \sum_{i=1}^{3} Ra_i \, \frac{\partial c_i'}{\partial \xi_1} \tag{S40 c}$$

$$\Delta \omega_3^* = 0 \tag{S40 d}$$

where $\vec{u^*}_\tau = (u_1^*, u_2^*, u_3^*)$ is the steady state velocity expressed in the dimensionless coordinates $\vec{\xi} = (\xi_1, \xi_2, \xi_3)$. The same Ansatz (S18) for the concentrations leads to the same secular equation (S19) for $\vec{\kappa}\vec{u}_\tau$, having the same solution (S20) and consequently the same expressions (S21 a,b) for the concentration amplitudes c_{0i}. The involved quantity $\vec{\kappa}\vec{u}_\tau$ has now the generalized meaning

$$\vec{\kappa}\vec{u}_\tau = \kappa_1 u_1 + \kappa_2 u_2 + \kappa_3 u_3 \tag{S41}$$

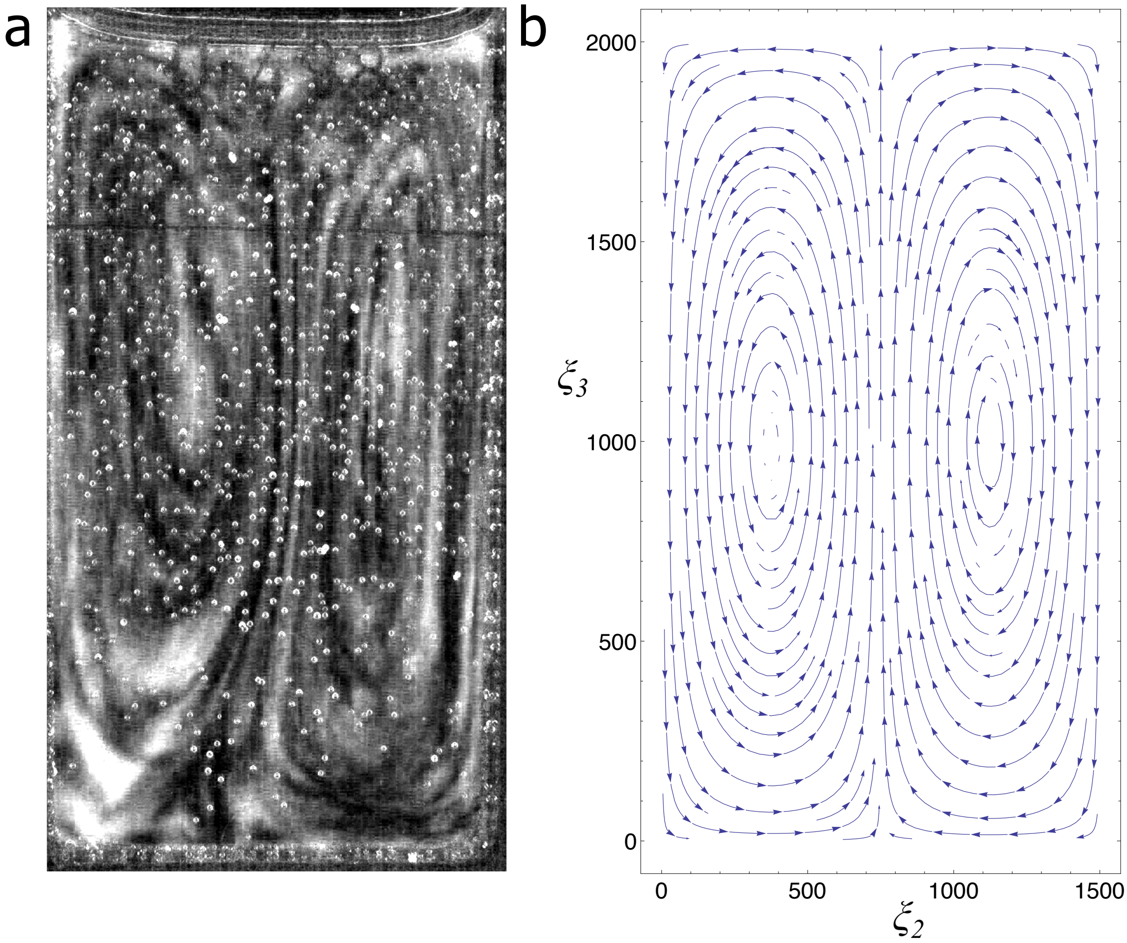

Figure S5. Comparison between experiment and theory, **(a)** shows the convection cells developed in a special cuvette in the chaotic phase (see also Fig. 2 A in the main article), **(b)** shows the calculated velocity field according to formula (S33).

and we posses no stream function Ψ more. Instead we have the three conponents of the velocity obeying the following differential equations:

$$\Delta\omega_1^* = -\sum_{i=1}^{3} Ra_i\, c_{0i}\, \kappa_2\, e^{\vec{\kappa}\vec{\xi}} \tag{S42 a}$$

$$\Delta\omega_2^* = \sum_{i=1}^{3} Ra_i\, c_{0i}\, \kappa_1\, e^{\vec{\kappa}\vec{\xi}} \tag{S42 b}$$

$$\Delta\omega_3^* = 0 \tag{S42 c}$$

The solutions of these equations are (we skip in the following the stars) given by

$$\omega_1 = \tilde{\chi}(\vec{\kappa})\,\kappa_2\, e^{\vec{\kappa}\vec{\xi}} \tag{S43 a}$$

$$\omega_2 = -\tilde{\chi}(\vec{\kappa})\,\kappa_1\, e^{\vec{\kappa}\vec{\xi}} \tag{S43 b}$$

$$\omega_3 = \text{const.} \tag{S43 c}$$

where now

$$\tilde{\chi}(\vec{\kappa}) = \frac{1}{4\pi}\sum_{i=1}^{3}|Ra_i|c_{0i}\int\limits_{-\frac{a}{2}}^{\frac{a}{2}}\int\limits_{-\frac{a}{2}}^{\frac{a}{2}}\int\limits_{-\frac{b}{2}}^{\frac{b}{2}}\frac{e^{-\vec{\kappa}\vec{\eta}}}{|\vec{\eta}|}\,d\eta_1 d\eta_2 d\eta_3 \tag{S44}$$

To regain the components of the velocity we apply the rotor operator on the definition of the vorticity

$$\vec{\omega} = \nabla\times\vec{u}$$

and use the fact that $\nabla\vec{u} = 0$ for an incompressible fluid. So we find

$$\Delta\vec{u} = -\nabla\times\vec{\omega} \tag{S45}$$

As $\vec{\omega}$ is already known form (S43) solving the Poisson equation (S45) we obtain:

$$\vec{u}(\vec{\xi}) = -\frac{1}{4\pi}\int\frac{\nabla_{\vec{\eta}}\times\omega(\vec{\eta})}{|\vec{\xi}-\vec{\eta}|}\,d\vec{\eta}. \tag{S46}$$

Using the representation of the velocity field, and of the vorticity by means of their Fourier transforms one may find another relationship between $\vec{u}$ and $\vec{\omega}$:

$$u_1(\vec{\xi}) = -\frac{1}{4\pi}\int\frac{\omega_2(\vec{\eta})}{|\vec{\xi}-\vec{\eta}|^2}\,d\vec{\eta} \tag{S47 a}$$

$$u_2(\vec{\xi}) = -\frac{1}{4\pi}\int\frac{\omega_1(\vec{\eta})}{|\vec{\xi}-\vec{\eta}|^2}\,d\vec{\eta}. \tag{S47 b}$$

The remaining coordinate of the velocity u_3 may be found integrating the equation $\omega_2 = \frac{\partial u_1}{\partial\xi_3} - \frac{\partial u_3}{\partial\xi_1}$:

$$u_3 = \int \frac{\partial u_1}{\partial \xi_3}\, d\xi_1 - \int \omega\, d\xi_1 + g(\xi_2, \xi_3) \tag{S48}$$

where $g(\xi_2, \xi_3)$ is an arbitrary function. These apperently redundant complications lead together with the fact that the fluid is incompressible to the results

$$\kappa_1 = 0 \quad \text{and} \tag{S49}$$

$$\vec{\kappa} \cdot \vec{u} = 0. \tag{S49 b}$$

Through (S49 b) we refound the old result given by (S34).
The condition that through the cuvette at least one flow circle takes place lead to the 'quantization' rules

$$\kappa_2 = \frac{i\pi n}{a} \qquad \text{with } n \text{ odd integer} \tag{S50 a}$$

$$\kappa_3 = \frac{i\pi m}{b} \qquad \text{with } m \text{ odd integer} \tag{S50 b}$$

Coming back to the representation (S46) and selecting form $e^{\vec{\kappa}\vec{\xi}}$ just

$$e^{\vec{\kappa}\vec{\xi}} = e^{\kappa_1 \xi_1}\ \sin(k_2\xi_2)\sin(k_3\xi_3) \tag{S51}$$

we obtain

$$(u_1, u_2, u_3) = \left(0,\ \frac{\tilde{\chi}(\vec{\kappa})\,\tilde{\phi}(\vec{\kappa})\,k_2 k_3 \sin(k_2\xi_2)\cos(k_3\xi_3)}{4\pi},\ -\frac{\tilde{\chi}(\vec{\kappa})\,\tilde{\phi}(\vec{\kappa})\,k_2^2 \cos(k_2\xi_2)\sin(k_3\xi_3)}{4\pi}\right). \tag{S52}$$

Here $k_2 = \frac{\pi n}{a}$, $k_3 = \frac{\pi m}{b}$ and the condition $\kappa_1 = 0$ was taken after performing the differential and integral computations in (S46).
The appearing factor $\tilde{\phi}(\vec{\kappa})$ is given by:

$$\tilde{\phi}(\vec{\kappa}) = \int_{-\frac{a}{2}}^{\frac{a}{2}} \int_{-\frac{a}{2}}^{\frac{a}{2}} \int_{-\frac{b}{2}}^{\frac{b}{2}} e^{-\kappa_1 \eta_1} \frac{\cos(k_2\eta_2)\cos(k_3\eta_3)}{|\vec{\eta}|}\ d\eta_1 d\eta_2 d\eta_3 \tag{S53}$$

We stress the fact that the solution (S52) is only a sufficient one. It is implied by (S48) which itself is also only a sufficient, but not necessary condition. However it is able to describe the dynamichs in the standard cuvette at least in the case where it is partially filled.

Because of the validity of the equation (S49 b) being identically with (S34) the chaos/order analysis remains qualitatively unchanged; only the formula of $\frac{c_3^*}{\epsilon}$ has a slightly different form:

$$\frac{c_3^*}{\epsilon} = \pi^2 \frac{1 + \delta_2 \pi^2 (\frac{n^2}{a^2} + \frac{m^2}{b^2})}{1 + f + \delta_2 \pi^2 (\frac{n^2}{a^2} + \frac{m^2}{b^2})} \cdot \left(\frac{n^2}{a^2} + \frac{m^2}{b^2} \right) \tag{S54}$$

REFERENCES

[1]J. Argyris, G. Faust, M. Haase, and M. Friedrich. *Die Erfoschung des Chaos*. Springer-Verlag, 2017.

[2]P. Glansdorf and I. Prigogine. *Thermodynamic theory of Structure, Stability and Fluctuations*. Wiley-Interscience, 1971.

[3]H. Kantz and T. Schreiber. *Nonlinear Time Series Analysis*. Cambridge University Press, 2004.

[4]L. Landau and E. Lifshitz. *Lehrbuch der Theoretischen Physik, Vol. VI, Hydrodynamik*. Harry Deutsch Verlag, 2007.

[5]Nadia Marchettini, Marcello Antonio Budroni, Federico Rossi, Marco Masia, Maria Liria Turco Liveri, and Mauro Rustici. Role of the reagents consumption in the chaotic dynamics of the Belousov–Zhabotinsky oscillator in closed unstirred reactors. *Physical Chemistry Chemical Physics*, 12(36):11062–11069, 2010.

[6]Federico Rossi, Marcello Antonio Budroni, Nadia Marchettini, Luisa Cutietta, Mauro Rustici, and Maria Liria Turco Liveri. Chaotic dynamics in an unstirred ferroin catalyzed Belousov-Zhabotinsky reaction. *Chemical Physics Letters*, 480(4-6):322–326, 2009.

[7]B. Saltzman. Finite amplitude free convection as an initial value problem - I. *Science*, 19(4):329–341, 1962.

[8]S. Scott. *Chemical Chaos*. Calderon Press, 1991.

[9]Xingbo Wang, Philips John Green, Jean-Baptiste Thomas, Jon Yngve Hardeberg, and Pierre Gouton. Evaluation of the Colorimetric Performance of Single-Sensor Image Acquisition Systems Employing Colour and Multispectral Filter Array. In Alain Treméau, Raimondo Schettini, and Shoji Tominaga, editors, *Proceedings of the 5th Computational Color Imaging Workshop*, volume 9016, 2015.

[10]M. A. Zayed and M. M. Taya. Spectrophotometric Study of the Reaction between Tryptophan and Ferroin at Different Forms. *Egypt J. Chem.*, 58:237–258, 2015.
